# A Review of Clinical Trials Involving Genetically Modified Bacteria, Bacteriophages and Their Associated Risk Assessments

**DOI:** 10.1089/apb.2024.0002

**Published:** 2024-12-16

**Authors:** Paul Gulig, Scott Swindle, Mark Fields, Daniel Eisenman

**Affiliations:** ^1^ Department of Molecular Genetics and Microbiology, University of Florida, Gainesville, Florida, USA.; ^2^ Advarra, Columbia, Maryland, USA.; ^3^ Department of Ophthalmology, Yale University, Yale School of Medicine, New Haven, Connecticut, USA.

**Keywords:** clinical trial, bacteria, phage, risk assessment, gene therapy, genetically modified organisms

## Abstract

**Introduction:**

Discussion of gene-modified investigational products (IPs) in clinical trials has largely focused on nucleic acid-based vectors, viral vectors, and gene-modified cellular products involving mammalian cells. Use of bacteria and bacteriophages as IPs is resurgent, and discussion of the risks associated with genetic modification of these organisms has become pertinent to the biosafety community.

**Methods:**

This review article summarizes the United States Food and Drug Administration classification for IPs comprising bacteria or bacteriophages and provides an overview of clinical trials conducted to date involving genetically modified bacteria. The risk assessment for bacterial or bacteriophage-based IPs is discussed.

**Conclusion:**

The risk assessment process for bacterial or bacteriophage-based IPs is different from that of gene expression vectors and mammalian cells. Greater consideration must be given to the attenuating mutations affecting virulence, replication competency, antibiotic susceptibility, and persistence in the environment. With the recent growth in clinical trials involving genetically modified bacteria, biosafety professionals and Institutional Biosafety Committees with responsibilities including oversight of clinical trials must become familiar with the associated risk assessment.

## Introduction

We previously described how the U.S. regulatory environment is evolving to accommodate a boom in gene therapy research.^1^ In the two most recent revisions to the National Institutes of Health (NIH) Guidelines for Research Involving Recombinant or Synthetic Nucleic Acid Molecules (NIH Guidelines), the NIH Office of Science Policy sequentially shifted regulatory oversight of human gene transfer research from the NIH Recombinant DNA Advisory Committee to the Food and Drug Administration (FDA).^2^ The FDA has issued numerous guidance documents intended for pharmaceutical companies developing investigational products (IPs) for clinical trials and designing clinical trials.

Many of these guidance documents bear relevance to biosafety professionals and Institutional Biosafety Committee (IBC) review. We previously discussed FDA guidance on clinical trials involving integrating vectors and gene editing technology as well as guidance on shedding and environmental impact for gene therapy products.^3,4^ In this review, we turn our attention to the quickly growing use of bacteria and their viruses, bacteriophages, as IPs in clinical trials and discuss the risks associated with genetically modified bacteria.

We begin this review article by summarizing the currently approved uses for bacteria and bacteriophages before delving into the FDA classification for IPs comprising bacteria or bacteriophages and providing an overview of clinical trials conducted to date involving genetically modified bacteria. The biosafety risk assessment process for bacterial or bacteriophage-based IPs is discussed. We will conclude by highlighting trends and future directions with potential to impact clinical research and the biosafety profession.

## Currently Approved Uses for Bacteria and Bacteriophages

The FDA has already started issuing approvals for bacterial products. In 2022, the FDA approved fecal microbial transplants for recurring *Clostridioides difficile* infections, and in April 2023, the FDA approved the first orally administered fecal microbiota-containing capsule for the prevention of recurrent *C. difficile* infection.^5^ Future applications of fecal microbial transplants range from bacterial dysbiosis, inflammatory diseases of the bowel, metabolic disorders, and even neurological or behavioral disorders.^6–8^

Research is ongoing to isolate a defined set of purified organisms to maximize purity, efficacy, and reproducibility, whereas minimizing risks from pathogenic organisms that may be present in bulk fecal microbiota transplants.^9^ Microbial contamination is an inherent problem for any human-derived product, such as organ transplants and even blood transfusions.^10–12^ In fact, as of 2020, there were five deaths from fecal microbiota transplants.^13^

Several countries have authorized the use of bacteriophages (phages) in applications ranging from agriculture, food production, and human health.^14^ The U.S. Environmental Protection Agency has approved phage products to combat Tomato bacterial canker, citrus canker, and Fire blight for apples and pears.^15–17^ Phage products are utilized in preharvest applications for livestock and poultry, as well as topical application for pets. There are several FDA-approved phage treatments as food additives to avoid foodborne illness in humans and pets.

Although phage therapy dates back to 1921, its use took a back seat to antibiotics with the discovery of penicillin in 1928 coupled with a lack of understanding of the limitations of phage therapy.^18^ Clinical trials involving phage-based IPs are resurgent with the emergence of antibiotic resistance as a serious medical threat. Such clinical trials focus largely on enteric bacteria, biofilm forming bacteria, and bacteria involved in skin infections.^19^

## Genetically Modified Bacteria

### Definitions

A summary of terms pertinent to genetically modified bacteria and bacteriophages is found in Table 1. The term “microbiome” was defined by Whipps 1988 as “a characteristic microbial community occupying a reasonably well-defined habitat, which has distinct physiochemical properties. The term thus not only refers to the microorganisms involved but also encompasses their theater of activity.”^20^ The related term “microbiota” represents the living organisms of the microbiome, that is, bacteria, protists, fungi, and algae.

**Table 1.** Definitions of Key Terms

Bacteriophage	A virus that infects and replicates within bacteria.
Horizontal gene transfer	Transfer of genetic information between organisms by means other than parent-to-offspring vertical transfer. The below three terms are modes for horizontal gene transfer.
Conjugation	Transfer of plasmids between bacteria by direct cell to cell contact.
Transformation	Uptake and chromosomal integration of exogenous DNA.
Transduction	Transfer of DNA between bacteria by bacteriophages.
Attenuation	Use of genetic modification to suppress or inhibit the proliferation, survival, or other biological activity of an organism, thereby mitigating the risks posed by that organism to its hosts and the environment. The below three terms are examples of attenuation mechanisms.
Replication-defective/conditionally replicative	The engineered inability of a biological organism to replicate or its ability to replicate only under defined conditions.
Virulence factor	Bacterial components that allow a bacterium to colonize its host and cause disease. Disruption of virulence factors by genetic modification can derisk the use of a bacteria for therapeutic means.
Auxotrophic	Inability of a bacteria to proliferate or survive in the absence of some exogenously provided essential substance. Engineering bacteria to be auxotrophic for an essential substance not found in humans is an attenuation strategy for therapeutic use of bacteria.
Live biotherapeutic products	A biological product that contains live organisms; is applicable to the prevention, treatment, or cure of a disease or condition of human beings; and is not a vaccine.^23^
Microbiome/microbiota	A community of microorganisms (e.g., fungi, bacteria, and viruses) that naturally resides in a particular habitat on the body (e.g., the gut and skin).
Probiotic	Live microorganisms that, when administered in adequate amounts, confer a health benefit to the host.^21^
Oncolytic	The ability of a virus or bacteria to preferentially infect and kill cancer cells.
Tumor microenvironment	The tissue local to the tumor comprised of solid tissue, immune effector cells, blood vessels, and signaling molecules. The tumor microenvironment is typically hypoxic, and its constituents support an immunosuppressive environment that allows the tumor to evade recognition and destruction by the immune system.
Hypoxic	Sub-physiological levels of oxygen in a tissue.
Obligate anaerobe	Bacteria that cannot survive in the presence of normal atmospheric concentrations of oxygen.
Facultative anaerobe	Bacteria that can survive in the presence of absence of normal atmospheric concentrations of oxygen.

Whether viruses, including bacteriophages, should be included in the microbiota is debated. Unfortunately, few people use these terms correctly, most often referring to the microbiota as the microbiome. In any case, this discussion is focused on organisms, specifically genetically modified organisms, not the theater of activity. Attempts at manipulating the microbiota with living organisms involve probiotics. According to the International Scientific Association for Probiotics and Prebiotics, “probiotics” are “live microorganisms that, when administered in adequate amounts, confer a health benefit on the host.”^21^

The benefits of probiotics have been known for thousands of years, even though the microbiological basis was not understood.^22^ As the analysis and development of probiotics increased exponentially, in 2016 the FDA published the Chemistry, Manufacturing, and Control guidelines for live biotherapeutic products (LBPs).^23^ According to that document, an LBP is “…a biological product that: (1) contains live organisms, such as bacteria; (2) is applicable to the prevention, treatment, or cure of a disease or condition of human beings; and (3) is not a vaccine. For the purposes of this document, LBPs are not filterable viruses, oncolytic bacteria, or products intended as gene therapy agents and, as a general matter, are not administered by injection.”

The document further defines recombinant LBPs as “a live biotherapeutic product composed of microorganisms that have been genetically modified through the purposeful addition, deletion, or modification of genetic material.” It is noteworthy that simply deleting genetic sequences is considered recombinant rather than addition or changing of sequences.

This document was aimed at guiding the submission of Investigational New Drug (IND) applications for LBPs, specifically the chemistry, manufacturing, and control information that is part of an IND application. It did not establish new regulations. When discussing candidates as probiotics or LBPs, the term Generally Regarded as Safe (GRAS) is important, as it exempts the item from much regulation. According to the FDA (www.fda.gov), “the use of a food substance may be GRAS either through scientific procedures or, for a substance used in food before 1958, through experience based on common use in food.”

Whether the substance is a food additive or food ingredient, determination of GRAS status falls on the FDA or experts outside of the government, respectively. According to the FDA rule, substances used before 1958 are deemed GRAS based on a safe history of use.

This discussion focuses on the development and regulation of genetically modified LBPs, most often administered as probiotics, as well as other recombinant bacteria and bacteriophages used to treat specific diseases including cancer or as vaccines. Unlike viruses, bacteria are typically not used to deliver genes to human cells, but there are some examples of this, such as the bacTRL-IL-12 system we will discuss later in this review. As such, this is not technically gene therapy, since human cell genetics are not being altered.

However, we will use this term for purposes of discussion since recombinant DNA is being introduced into humans. The overview of clinical trials toward the end of this review article will summarize how genetically modified bacteria are being developed to treat genetic diseases, diseases without a known genetic element, and cancer. There are no FDA-approved recombinant bacterial therapies for humans, although a recombinant LBP is being marketed in the United States.^24^ In addition, there is one recombinant live attenuated bacterial vaccine, Vaxchora for cholera.^25^

### From Pathogens to Therapies

Understanding the roles of bacteria in causing human disease dates back to the days of Louis Pasteur, Robert Koch, and other fathers of bacteriology. Not long after specific bacterial species were identified as the causative agents of specific diseases, they were developed into vaccines. For example, Louis Pasteur created a vaccine strain of *Bacillus anthracis* to prevent anthrax.^26^

More recently, as the ability of certain pathogens to stimulate different arms of the immune system (e.g., mucosal immunity, humoral immunoglobulin, and cell-mediated immunity) was discovered, along with an understanding of the critical virulence factors and physiology required for production of disease, mutant strains were created to serve as live-attenuated vaccines that could deliver recombinant antigens from other pathogens.^27^ As noted earlier, The FDA has excluded vaccines from the definition of LBPs. However, the same advances in attenuating bacterial pathogens for vaccine development are also important for biosafety and biocontrol of LBPs.

### Bacteria as LBPs

The ability to engineer bacteria with a seemingly limitless variety and number of transgenes as well as their coexistence with humans make them attractive candidates as gene therapy vectors. Some examples of current developments are providing beneficial or missing metabolic functions to alleviate disease, providing immunomodulatory factors to alleviate autoimmune diseases, fighting cancer by stimulating the tumor microenvironment immune response and presenting tumor-specific antigens, or increasing effectiveness of chemotherapy.^28^

Usually, bacterial gene therapy in the form of recombinant LBPs consists of genetically engineered probiotics that colonize the body. Unlike viruses, bacteria are rarely going to be administered parenterally, that is, injected, but there are exceptions, as noted hereunder. Parenteral administration is difficult because, unlike viruses that are rarely toxic or inflammatory by their chemical nature, bacteria contain highly inflammatory cellular components known as pathogen-associated molecular patterns (PAMPs) that trigger cellular reactions from pathogen recognition receptors expressed by host cells.

Examples of PAMPs include lipopolysaccharide, peptidoglycan, and flagellar proteins. Many viruses, in contrast to most bacteria, have evolved mechanisms that prevent the stimulation of a vigorous adaptive immune response. Therefore, parenteral administration of live bacteria would likely result in their clearance through an immune response.

### Bacteria as Vaccines Against Infection and Cancer or as Oncolytic Agents

There are two live attenuated bacterial vaccines that are FDA-approved: Vaxchora for cholera and Vivotif for typhoid fever. Vaxchora was the first and is the only recombinant bacterial strain to receive FDA approval for use in humans. It is possible that it will be followed by other genetically engineered vaccines for enteric infections. The most likely uses of live attenuated bacterial vaccines would be in the gastrointestinal system, because it is rich with microbiota and can tolerate the introduction of foreign microorganisms better than the respiratory tract. However, live recombinant bacteria are also being investigated for use on the skin (Table 2).

**Table 2.** Clinical use of genetically modified bacteria for non-cancer indications

***Disease***	***Species***	***Strain***	***Route***	***Phase***	***Clinical trial No.***	***Attenuation mechanism (transgene)***	***Ref.***
Inflammatory bowel disease	*Lactococcus lactis*	AG014	Oral	I	2014-000190-39^a^	(Tumor necrosis factor antibody)	—
Crohn's disease	*L. lactis*	LL-Thy12	Oral	I	—	Thymidine auxotrophy: *ΔthyA* (Interleukin-10)	^89^
Ulcerative colitis	*L. lactis*	AG011	Oral	II	NCT00729872	(Interleukin-10)	
Celiac disease	*L. lactis*	AG017	Oral	IND	—	(Gliadin peptide and Interleukin 10)	—
Diabetes mellitus	*L. lactis*	AG019	Oral	II	NCT03751007	(Proinsulin; interleukin-10)	^91^
Cirrhosis and hyperammonemia	*Escherichia coli* (Nissle)	SYNB1020	Oral	II	NCT03447730	Thymidine auxotrophy: *ΔthyA* (enzymes for consumption and metabolism of ammonia)	^96^
Enteric hyperoxaluria	*E. coli* (Nissle)	SYNB8802	Oral	I	NCT04629170	Thymidine auxotrophy: *ΔthyA* (enzymes for consumption and metabolism of oxalate)	^97^
Enteric hyperoxaluria	*Bacteroides vulgatus*	NOV-001	Oral	II	NCT04909723	Porphyran-dependent expression of arginine tRNA ligase (enzymes for consumption and metabolism of oxalate)	—
Phenylketonuria	*E. coli* (Nissle)	SYNB1934	Oral	III	NCT05764239	DAP auxotrophy: *ΔdapA* (enzymes for consumption and metabolism of phenylalanine)	^123^
Netherton syndrome	*Staphylococcus epidermidis*	ATR-12	Topical	I	NCT06137157	d-Alanine auxotrophy: Δ*alr/Δdat* (SPINK5)	—
Skin appearance	*S. epidermidis*	AZT-04	Topical	I	NCT03820076	d-Alanine auxotrophy: Δ*alr/Δdat* (no transgene)	—
Oral mucositis	*L. lactis*	AG013	Topical	II	NCT03234465	Thymidine auxotrophy: *ΔthyA* (Trefoil factor 1)	^90^
POC studies in healthy subjects	*Listeria monocytogenes*	BMB54	Oral	I	NCT01311817	*ΔactA/ΔinlB* (POC antigen)	^124^
POC studies in healthy subjects	*L. monocytogenes*	BMB72	Transcutaneous	I	NCT01311817	*ΔactA/ΔplcB* (POC antigen)	^124^

The table lists clinical trials evaluating use of genetically modified bacteria to treat non-cancer diseases.

Clinical trial number refers to the identification code of each clinical study upon registration at ClinicalTrials.gov

^a^ EudraCT (European Union Drug Regulating Authorities Clinical Trials Database) clinical trial number.

*ΔactA*, disruption of the actin assembly-inducing protein gene inhibits cell to cell spread through actin-based motility; *ΔinlB*, disruption of the internalin B gene inhibits entry into nonphagocytic cells; *ΔplcB*, disruption of the phospholipase C gene inhibits the bacterium's vacuole release into the cytosol; *Δalr/Δdat*, alanine racemase and d-amino acid transaminase gene disruptions; *ΔdapA,* 4-hydroxytetrahydropicolinate synthase gene disruption; *ΔthyA*, thymidine synthase gene disruption.

IND, investigational new drug application; POC, proof of concept.

Considerable research is being done on using recombinant bacteria as cancer vaccines to immunize cancer patients against their tumors or to kill the tumor cells directly as oncolytic therapy.^29^ Two species being examined for the latter are *Salmonella enterica* serovar Typhimurium (*Salmonella* Typhimurium) and *Listeria monocytogenes*, both of which can invade tumor cells and induce lysis or apoptosis.

## Special Challenges and Considerations of Recombinant Bacterial LBPs

### Replication

A challenge to using bacteria as LBPs is biocontainment, restricting where the bacteria can migrate, colonize, and grow. There are a few cases where bacteria are administered parenterally and are unlikely to exit the treated research subject. However, most LBPs are going to be administered to colonize mucosal surfaces, primarily the gastrointestinal tract, and will therefore exit the body. The release of recombinant organisms into the environment, including the sewage treatment system, is a biosafety concern for both public health and environmental protection.

Therefore, most bacterial LBPs are going to be engineered to be conditionally replicative. It is relatively easy to create replication-defective viruses by deleting critical nucleic acid replication genes. These replication-defective viruses can be produced using specialized host cells that complement by providing the missing genes required for replication in trans. Such replication-defective viruses can serve as single-pass gene delivery vectors.

However, the complexity of bacterial physiology with regard to replication makes bacteria more difficult to attenuate for replication. For example, they cannot be simply grown in permissive host cells that phenotypically complement missing functions. Instead, genes for the production of essential compounds are deleted, and the bacteria are then grown in specialized media that provide the missing compound. This presents the problem that the missing compound cannot be present in the human body or possibly the environment, or the bacteria would grow in an uncontrolled manner.

**Figure 1.**
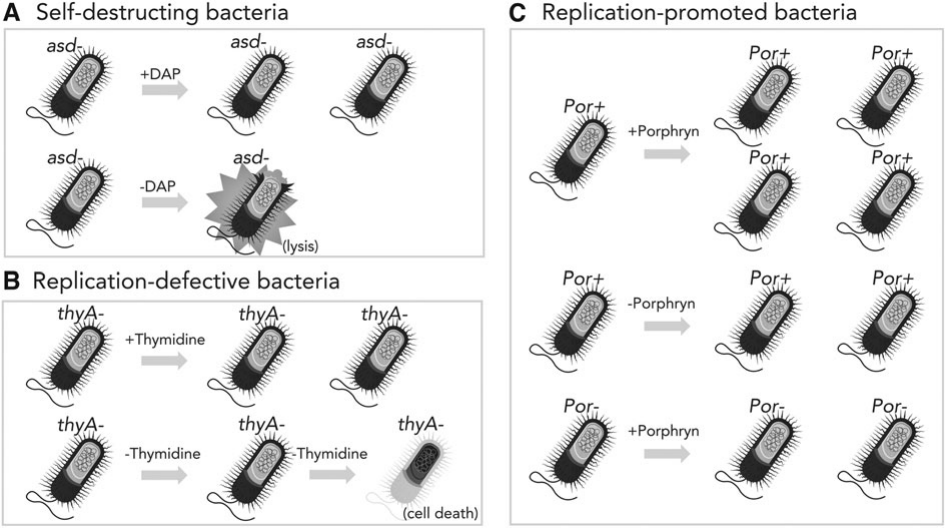
Genetic control of bacterial survival and replication. Mechanisms by which genetics of bacteria can be manipulated to prevent unwanted survival or growth, to promote growth, or to select for maintenance of recombinant plasmids are illustrated. **(A)** Self-destructing bacteria. The *asdA* gene is deleted from the chromosome (*asdA*^−^). If the bacteria are not provided with DAP in their environment, they will lyse because of loss of peptidoglycan integrity. **(B)** Replication-defective bacteria. The *thyA* gene is deleted from the chromosome. If the bacteria are not provided with thymidine in their environment, they cannot replicate because of the inability to make DNA, and they will eventually die. **(C)** Replication-promoted bacteria. The bacteria are endowed with the genes encoding the ability to metabolize porphyrin, an algal seaweed polysaccharide. Por+ bacteria can replicate more robustly when provided with porphyrin in the environment. *Por*^−^ bacteria can replicate, but do not experience the growth boost. DAP, diaminopimelic acid.

A common strategy for controlling replication, growth, and survival is disruption of genes necessary for synthesis of the peptidoglycan containing cell wall of gram-negative bacteria (Figure 1). *asd* and *dapA* encode for enzymes (aspartate dehydrogenase and 4-hydroxytetrahydropicolinate synthase, respectively) essential for the production of diaminopimelic acid (DAP), a key intermediate in peptidoglycan synthesis. Absence of DAP causes the cell to lyse (Figure 1A), called DAPless death, much in the same way as if it was treated with a β-lactam antibiotic such as penicillin.^30^ Other than bacteria, the only other known source of DAP is some plants.

Therefore, *asd* or *dapA* mutant bacteria will die very quickly in the body or environment. d-Alanine is another compound necessary for peptidoglycan formation. Bacteria disrupted for both *dal,* encoding an alanine racemase, and *dat,* encoding a d-amino acid aminotransferase, are unable to survive in the absence of exogenous d-alanine, which is found only in trace quantities in vertebrate animals. The Δ*dal*/Δ*dat* strains are among the most common developed as tumor vaccines that have already been evaluated in human clinical trials (Table 3).^31^ Use of bacteria in oncology trials and other diseases indications will be discussed later in this review.

**Table 3.** Clinical use of genetically modified bacteria in cancer

***Disease***	***Species***	***Strain***	***Route***	***Phase***	***Clinical trial No.***	***Attenuation mechanism (transgene/immunogen)***	***Ref.***
HPV-positive cancers (cervical, anal, rectal, lung, head and neck)	*Listeria monocytogenes*	ADXS11–001	IV/IT	I–III	NCT02164461, NCT01266460, NCT01671488, NCT02399813, NCT02531854, NCT02002182, NCT02853604, NCT02291055	*ΔprfA* (HPV-16 E7)	^100,125–129^
Her2-positive cancers	*L. monocytogenes*	ADXS31–164	IV	I–II	NCT02386501	*ΔactA; Δdal/Δdat* (HER2/neu)	—
Prostate cancer	*L. monocytogenes*	ADXS-504	IV	I	NCT05077098	*ΔactA; Δdal/Δdat* (24 common TAAs)	^130^
Prostate cancer	*L. monocytogenes*	ADXS31–142	IV	I–II	NCT02325557	*ΔactA; Δdal/Δdat* (prostate cancer antigen)	^131^
Lung cancer	*L. monocytogenes*	ADXS-503	IV	I–II	NCT03847519	*ΔactA; Δdal/Δdat* (common TAAs)	^132^
Metastatic cancers	*L. monocytogenes*	ADXS-NEO	IT	I	NCT03265080	*ΔactA; Δdal/Δdat* (patient-specific TAAs)	^133^
Lung cancer	*L. monocytogenes*	JNJ-64041757	IV	I–II	NCT03371381, NCT02592967	*ΔactA/ΔinlB* (EGFRvIII, mesothelin)	^104^
Prostate cancer	*L. monocytogenes*	JNJ-64041809	IV	I	NCT02625857	*ΔactA/ΔinlB* (common TAAs)	^134^
Advanced/metastatic tumors and lymphoma	*Escherichia coli* (Nissle)	SYNB1891	IT	I	NCT04167137	*ΔdapA; ΔthyA* (diadenylate cyclase)	^98^
Solid tumors	*Bifidobacterium longum*	bacTRL-IL-12	IV	I	NCT04025307	Obligate anaerobe (interleukin-12 plasmid)	—
Mesothelin-positive cancers (pancreatic, mesothelioma, ovarian/fallopian, and gastroesophageal)	*L. monocytogenes*	CRS-207	IV	I–II	NCT00327652, NCT00585845, NCT02004262, NCT01417000, NCT02243371, NCT03190265, NCT03006302, NCT02575807, NCT03122548, NCT01675765, NCT03175172	*ΔactA/ΔinlB* (mesothelin)	^135–141^
Colorectal cancer	*L. monocytogenes*	pLADD	IV	I	NCT03189030	*ΔactA/ΔinlB* (patient-specific TAAs)	—
Multiple myeloma	*Salmonella* Typhimurium	TXSVN	IV	I	NCT03762291	*ΔpurD/ΔhtrA* (survivin)	^142^
Solid tumors	*Salmonella* Typhimurium	TAPET-CD	IT	I	—	*ΔpurI/ΔmsbB* (cytosine deaminase)	^108^
Solid tumors	*B. longum*	APS001F	IV	I–II	NCT01562626	Obligate anaerobe (cytosine deaminase)	^92^
Advanced or metastatic tumors	*Salmonella* Typhimurium	VNP20009	IV	I	NCT00004988, NCT00006254, NCT00004216	*ΔpurI/ΔmsbB* (No transgene)	^143^
Glioblastoma	*L. monocytogenes*	ADU-623	IV	I	NCT01967758	*ΔactA/ΔinlB* (EGFRvIII, NY-ESO-1)	^144^
Familial adenomatous polyposis	*E. coli*	CEQ508	Oral	I	—	(β-catenin shRNA)	^145^
Liver cancer pancreatic cancer	*Salmonella* Typhimurium	SalpIL2, Saltikva	Oral	I–II	NCT01099631, NCT04589234	Facultative anaerobe (interleukin-12)	^146,147^
Glioblastoma and pancreatic cancer	*Salmonella* Typhi	VXM01	Oral	I–II	NCT03750071, NCT02718443, NCT01486329, NCT01486329	Chemical mutagenesis (vascular endothelial growth factor receptor-2)	^106,148,149^
Neuroblastoma	*Salmonella* Typhimurium	SS2017	Oral	I	NCT04049864	Facultative anaerobe (patient-specific TAAs)	—

The table lists clinical trials evaluating use of genetically modified bacteria to treat cancer.

Clinical trial number refers to the identification code of each clinical study upon registration at ClinicalTrials.gov

*ΔactA*, disruption of the actin assembly-inducing protein gene inhibits cell to cell spread through actin-based motility; *Δdal/Δdat*, disruption of the alanine racemase and d-amino acid transaminase genes results in d-alanine auxotrophy; *ΔdapA,* disruption of the 4-hydroxytetrahydropicolinate synthase gene results in DAP auxotrophy; *ΔhtrA*, disruption of the *htrA* serine protease gene diminishes the bacterium's stress response systems; *ΔinlB*, disruption of the internalin B gene restricts entry to nonphagocytic cells; *ΔmsbB*, disruption of the *msbB* myristoyl transferase gene reduces toxicity of lipopolysaccharide; *ΔprfA*, disruption of the gene encoding the *prfA* transcription factor essential for expression of the bacterium's virulence factors; *ΔpurD,* disruption of the *purD* gene results in adenine auxotrophy; *ΔpurI*, disruption of the *purI* AIR synthetase gene results in purine auxotrophy; *ΔthyA*, disruption of the thymidine synthase gene results in thymidine auxotrophy.

DAP, diaminopimelic acid; HPV, human papilloma virus; IT, intratumoral; IV, intravenous; shRNA, small hairpin RNA; TAA, tumor-associated antigen.

Disruption of the *thyA* gene encoding thymidylate synthase necessary for production of thymidine and, therefore, DNA is another common attenuation strategy.^32–34^ Freely available thymidine is not found in significant amounts in the body or environment, but it is not as scarce as DAP. Furthermore, lack of thymidine prevents replication, but does not necessarily lead to rapid death (Figure 1B), unlike the case for DAP. A detriment to DAPless death or thymidine starvation is preventing the recombinant LBP from doing its job.

If the bacteria die too quickly or are unable to grow to achieve an effective critical mass in the body, they will be unable to carry out their intended function. In contrast, the lysis of the bacterial cell by DAPless death can enable the release of recombinant cellular enzymes to achieve some beneficial end. This has already been engineered into strains to treat hyperoxaluria (excessive oxalate) and phenylketonuria (inability to metabolize phenylalanine).

A different approach to creating conditionally replicative bacteria is to place key genes for growth and replication under promoters so that the genes are only expressed under specific conditions that can be controlled in both the laboratory, human host, and environment. For example, placing the *asd* gene under control of the *araBAD* promoter allows the growth and survival of the bacteria only when arabinose is present.^35^ Arabinose is not normally present in the human body and is not widespread in the environment. Another regulatable system involves use of porphyrin, an algal polysaccharide, as a carbon source (Figure 1C). Bacteria capable of metabolizing porphyrin express genes required for its degradation when porphyrin is detected.

Some recombinant bacterial gene therapy products use porphyrin-induced expression systems to regulate both transgene expression and bacterial growth.^36^ A problem with using regulatable promoters to control replication is that bacteria can mutate to circumvent this control. It is a sine quo non that when bacteria are faced with a fitness challenge, they often respond with a mutation to overcome the challenge. This is all too well known in the development of new antibiotics, which remain effective for brief periods until resistance arises. However, with careful design, the likelihood of compensatory mutations can be minimized.

Another manipulation of bacterial replication is aimed at promoting the specific growth of bacteria in a tumor environment, which is hypoxic and enriched for certain nutrients, including amino acids, as opposed to healthy tissues where the bacteria are of no benefit.^29^ *Salmonella* Typhimurium A1 is auxotrophic for leucine and arginine.^37^ It has shown efficacy in numerous mouse models of human cancers but has not been examined in clinical trials. SF104 is *Salmonella* Typhimurium that is deleted for the *aroA* gene, resulting in an aromatic compound auxotrophy, added to two other mutations affecting lipopolysaccharide biosynthesis.^38^ The *aroA* mutation unexpectedly increased the antitumor activity of the strain, over and above the intended auxotrophy.

*Salmonella* Typhimurium VNP20009 is deleted for the *msbB* and *purI* genes.^39^ *msbB* is involved with lipopolysaccharide biosynthesis, and *purI* is involved with adenine synthesis. VNP20009 was studied in clinical trials, but results were disappointing.

As noted earlier, manipulation of the essential gene *asd* is used as a mechanism for controlling replication and survival. In *Salmonella* Typhimurium YB1, the *asd* gene was placed under a hypoxia-inducible promoter so that in the hypoxic tumor microenvironment, the bacteria would survive, but in normal tissues, the *asd* gene would not be expressed and the bacteria would lyse.^40^ This strain also has an *aroA* deletion making it an aromatic compound auxotroph. It has not been examined in clinical trials. These examples demonstrate how detailed knowledge of bacterial physiology is required to oversee the use of strains that have been engineered with very specific growth characteristics in the human host.

### Genetic Exchange

Over and above the issues with potentially replicating bacteria for administration to humans, other aspects of bacterial physiology and genetics present biological and regulatory challenges. The ability of bacteria to undergo horizontal gene transfer through a variety of mechanisms is problematic, as the recombinant DNA could find its way into other bacteria. Plasmids have been the workhorses of genetic engineering in bacteria since the earliest days of recombinant bacteriology. Plasmids can be transferred among diverse bacteria by conjugation, which requires cell–cell contact.

However, not all plasmids can be transferred by conjugation, so-called conjugative or auto-transmissible plasmids. Clearly conjugative plasmids are inappropriate for introducing recombinant DNA into bacterial LBPs. Mobilizable plasmids can be moved by conjugation only if the bacterial cell possesses a conjugative plasmid. Use of mobilizable plasmids is undesirable, as bacterial strains lacking conjugative plasmids could acquire them from other members of the microbiota. Therefore, nonconjugative plasmids are optimal.

However, even their use presents problems. First, their release into the extracellular milieu upon the death and lysis of the host bacteria cell could enable other bacteria to acquire the plasmid through transformation, that is, the uptake of naked DNA. Not all bacteria are able to perform natural transformation, and those that do usually only take up DNA from the same species. Another problem with plasmids is that they can be lost from the host cell by not replicating or partitioning into the progeny cells upon cellular division.

Therefore, essentially all plasmids encode selectable genetic markers so that only those bacteria that have retained the plasmid will grow under the appropriate conditions. The default selectable markers have been antibiotic resistance genes. The use of such genes in the laboratory is nearly universal, subject to some regulation to prevent genes for resistance to clinically relevant antibiotics being placed into certain pathogens. However, adding antibiotic-resistance genes into bacteria destined for use in the human body is undesirable, as these genes could find their way into other bacteria in the resident microbiota or environment.

**Figure 2.**
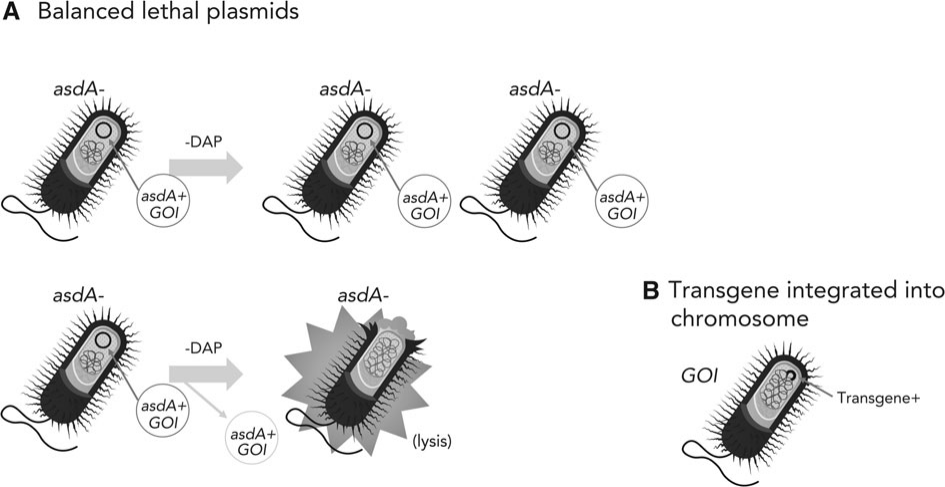
Maintenance of transgenes. Ensuring that the recombinant bacteria continue to carry the transgene through replication is important for efficacy. **(A)** Balanced lethal plasmids. Using antibiotic resistance genes as selectable markers for recombinant plasmids in bacteria that could be used in humans, animals, or plants is considered a risk for spreading antibiotic resistance. As for self-destructing bacteria, the *asdA* gene is deleted from the chromosome. The *asdA* gene is cloned into a recombinant plasmid encoding the GOI. As long as the plasmid is in the *asdA*^−^ bacteria, they can grow in the absence of DAP. If the plasmid is lost, the bacteria will lyse in the absence of DAP. **(B)** Integration of the transgene (GOI) into the bacterial chromosome. By integrating the gene of interest into the chromosome, the necessity of using plasmids is eliminated. The gene of interest can be integrated into the bacterial chromosome in several different manners. Allelic exchange recombinant can insert the gene of interest in any locus that will tolerate such an insertion, that is, a nonessential gene. Transposons can randomly insert the gene into the chromosome or, conversely, some transposons have preferred hotspots for integration. Certain bacteriophages can lysogenize specific bacteria, integrating into specific sites in the chromosome. A disadvantage of this method is that bacteriophages can promote recombination between bacterial cells, and that would not be acceptable. GOI, gene of interest.

A solution to this problem would be use of selectable markers based on properties other than antibiotic resistance. One such workaround is to delete a critical gene for growth and/or survival from the chromosome and then provide that gene on the recombinant plasmid. As long as the plasmid is in the host bacterial cell, it will complement the lethal mutation. However, once the plasmid is lost, the bacterium will fail to grow or will die. This system is called the balanced lethal system.^30^ Two such genes that are commonly used for balanced lethality are *asd* and *thyA* discussed earlier in terms of controlling bacteria replication and growth (Figure 2A).

To circumvent the use of plasmids entirely, the recombinant DNA can be inserted into the chromosome of the host strain (Figure 2B). This can be done using a variety of means, including allelic exchange recombination, transposable elements, or even bacteriophages. Not only is the recombinant DNA stable, but also the need for antibiotics is precluded.

However, even chromosomal DNA sequences can find their way into other bacteria through transformation or the third mechanism of horizontal gene transfer, transduction. Transduction involves viruses of bacteria, bacteriophages, to move DNA from a donor cell to a recipient cell. Bacteriophages are ubiquitous in the environment and in the human body, so transduction is a real concern. However, bacteriophages are extremely specific in their host ranges, so they could only transfer DNA among select strains of the same bacterial species.

### Risk/Attenuation of the Bacterial Host Strain

In contrast to the study of viruses as gene therapy agents for ∼40 years, the use of bacteria for this purpose is relatively new. As is the case for any change in a well-established system, challenges arise. The majority of studies using bacteria as gene therapy agents and cancer treatments utilize commensal organisms of the human microbiota or other bacteria that are GRAS. As such, the review of their safety rests primarily on the transgenes. However, as noted hereunder, there are several examples of bacterial pathogens being developed for gene therapy, particularly for treatment of cancer.

The oversight of these agents requires an understanding of the pathogenesis of the wild-type (WT) organism, particularly its virulence factors, as well as the nature of the attenuating mutations. The earliest manipulation of bacterial genomes to attenuate virulence involved nonrecombinant means, for example, chemical mutagenesis. This is exemplified in the *Salmonella enterica* serovar Typhi (*Salmonella* Typhi) vaccine strain, Ty21a, discussed hereunder. *Salmonella* Typhi, which is not endemic to the United States, causes typhoid fever, a systemic infection, in contrast to *Salmonella* Typhimurium, which causes localized gastroenteritis and is endemic to the United States.

Today, virulence genes are deleted using a variety of recombinant mechanisms. However, as noted earlier, simply restricting the growth or survival of vaccine strains cannot assure attenuation. First-in-human trials are particularly problematic, since preclinical animal results do not always translate to the human experience. A particularly notable example was the early attempt at creating a live attenuated vaccine for typhoid fever caused by *Salmonella* Typhi in the 1980s. Deletion of the *galE* gene that prevented the synthesis of lipopolysaccharide in the absence of exogenous galactose resulted in severe attenuation in mouse models. However, the g*alE* deletion mutant strain was virulent when tested in human clinical trials.^41^

Some bacterial pathogens do not have effective animal models. Another example of a live attenuated vaccine development failure was for *Vibrio cholerae* that causes cholera. It was known that cholera toxin is the primary virulence factor causing the life-threatening diarrhea, so it was expected that a strain mutated for the cholera toxin activity would be benign. There were no animal models to test this hypothesis. When live attenuated cholera toxin mutant vaccine strains were tested in humans, residual enteric toxicity in the form of diarrhea was noted. This led to the identification of numerous accessory toxins that contribute to pathogenesis of cholera.^42^ There are now FDA-approved safe and effective live attenuated vaccines for cholera (Vaxchora) and typhoid fever (Vivotif).

Finally, consideration for researchers, study subjects, and contacts is critical. Some attenuated strains of bacterial pathogens are safe for otherwise healthy individuals but are lethal for compromised people. Compromised does not always mean immunocompromised.

A tragic case in 2009 was the death of an investigator handling a vaccine strain of the causative agent of plague, *Yersinia pestis*.^43^ The KIM D27 strain is attenuated because of a mutation in the pigmentation genes; however, the investigator had a condition called hemochromatosis resulting in excess iron in the blood. The pigmentation system is involved with iron uptake, so the hemochromatosis rendered him hypersusceptible to infection with iron-starved organisms.

## Risk Assessment for Clinical Trials Involving Genetically Modified Bacteria

### Microbial Risks

**Figure 3.**
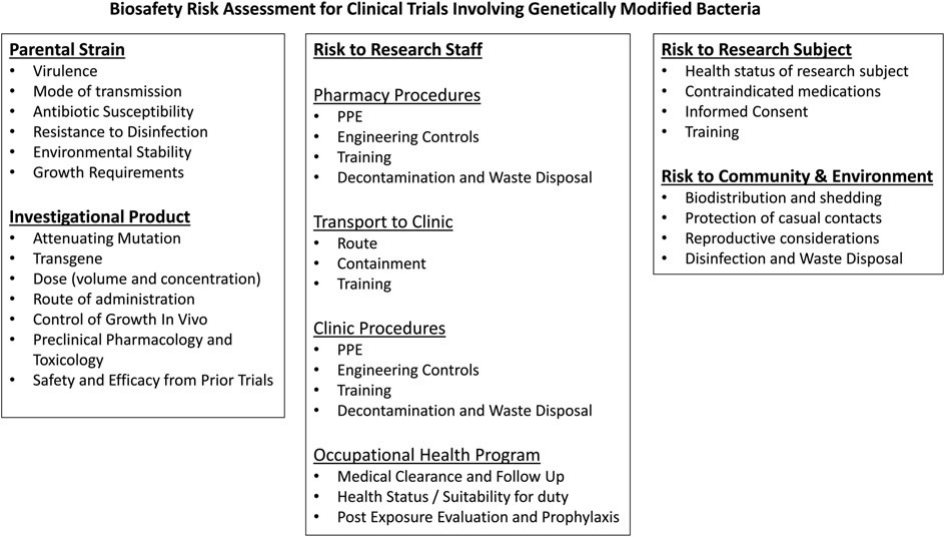
Components of a biosafety risk assessment for clinical trials utilizing genetically modified bacteria.

The risk assessment should consider risks to research subjects, research staff, as well as the community and environment (Figure 3). Assessment of the risks associated with a genetically modified bacterial IP begins with an understanding of the risks associated with the parental organism, including the virulence, mode of transmission, and growth requirements. The ability to treat exposures (antibiotic susceptibility) and mitigate environmental risks (resistance to disinfection, environmental stability, and release of antibiotic resistance genes into the environment) must also be considered.

The risks associated with the IP builds on the assessment of the parental organism by considering attenuating mutations and the function of the transgene. When considering the attenuation, one must assess whether it can revert to the earlier, more virulent phenotype or if it can regain virulence under any circumstances such as the health status of an especially susceptible host.^43,44^ The function of the transgene must also be considered for its ability to increase the virulence of the IP as well as its function within the host who may be in suboptimal health or immune compromised.

If the IP has been used in prior clinical trials, the safety and efficacy data from those trials should be evaluated. If the clinical trial under review constitutes a first-in-human experience, the risk assessment should include review of the preclinical pharmacology and toxicology data. Although clinical protocols, investigator brochures, and published studies focus on subject safety, some occupational safety information may be present in the IPs pharmacy manual and safety data sheet as well as the product insert for FDA-approved drugs.

A review of gene therapy studies found the route of administration determines which sites within the host will receive the greatest exposure. Sites with the greatest extent of biodistribution and likely shedding were anatomically downstream from the inoculation site.^45^ Although the article focused on viral vectors these concepts likely apply to bacteria.

However, the risk assessment must also consider whether the bacteria are motile and where they are likely to colonize within the host. The growth requirements of the organism should be considered along with any mutations affecting the ability of the organism to colonize and grow within the host. The IBC should ensure the Principal Investigator provides adequate training to research personnel including but not limited to the following:

- Awareness of the hazards associated with the research
- How to perform their duties safely (including personal protective equipment [PPE] and safety practices)?
- How personnel could be exposed?
- Disinfection and waste disposal practices
- Incident and spill response procedures
- Shipping (if appropriate).

### Occupational Health Considerations

Research involving bacteria poses an occupational exposure risk to research personnel.^46–50^ A survey of laboratory-acquired infections (LAIs) and pathogen escapes worldwide between 2000 and 2021 found bacteria account for 77% of LAIs.^51^ Medical evaluation by an occupational health provider can determine fitness for duty to conduct research involving infectious agents.

In our opinion, aspects to consider include, but are not limited to, age, health status, ability to use a respirator (if needed), and use of medications that may increase susceptibility to infection. Individuals with altered immune status, including those who are immune compromised, exhibit autoimmunity or on immune suppressive medication are more susceptible to infection.^52–57^ Individuals with pre-existing conditions may serve as hospitable hosts to organisms that may otherwise be considered unable to colonize or are avirulent to a healthy human host.^58–65^

Women who may be pregnant may opt to notify their physician of the nature of the research to determine if they should require additional protective measures or seek reassignment of duties. Risks of bacterial infection during pregnancy are well established.^66^ Maternal gut bacteria promote neurodevelopmental abnormalities in mouse offspring and bacterial infections have been linked to schizophrenia in humans.^67–71^ *L. monocytogenes* and *Treponema pallidum* are prominent bacterial fetal pathogens. *L. monocytogenes* is an intracellular pathogen commonly used in basic science research^72–75^ to test cell-mediated immunity and has been used extensively in clinical trials (Tables 2 and 3).

The IBC and/or occupational health program may need to consider how potential exposure to the IP may cross-react with tests for other organisms. For example, exposure to the Bacillus Calmette-Guerin vaccine derived from *Mycobacterium bovis* may result in false positive^76^ skin tests indicating exposure to *Mycobacterium tuberculosis*.

If an individual exhibits symptoms of exposure, the IP may be confirmed as the causative agent versus a community acquired WT organism by genetic tests of clinical isolates or specimens for the presence of the transgene or other genetic modifications. Whole genome sequencing or, less likely, pulse field gel electrophoresis may also be utilized to assess relatedness of WT strains to clinical isolates.

In our opinion, it is beneficial for IBCs at institutions to have pre-existing, collaborative relationships with their occupational health providers to proactively address issues involving occupational safety of research staff. Familiarity between the IBC and the occupational health provider also facilitates more effective incident response, associated postexposure care and reporting, if necessary.

### Risk Mitigation in the Clinical Setting

When assessing risks to research staff, it is necessary to consider the facilities, product handling procedures, and personnel that will be involved throughout the life of the IP. The risk mitigation plan must include training on the hazards posed by the IP as well as the engineering controls, PPE, and safety practices that cater to the needs of the various personnel roles without disrupting clinical operations or increasing risks to non-research personnel. The risk mitigation plan should include an incident response plan covering spills, exposures, and environmental releases. An occupational health program should be available to assess each employee's health status and suitability for duty as well as post-exposure evaluation, prophylaxis, and follow-up.

Receipt of the IP, storing, and dispensing typically take place at a pharmacy. Biosafety in the pharmacy setting was reviewed elsewhere.^77,78^ It is important to note that pharmacies may vary drastically based on their location and types of drugs they typically dispense. Investigational pharmacies in large academic medical centers are typically designed with U.S. Pharmacopeia 797 and 800 compliant facilities containing isolated dispensing rooms equipped with biosafety cabinets.^79,80^

In contrast, pharmacies in small satellite clinics may be as simple as small locked drug storage rooms with benchtops for dispensing. If biosafety cabinets are not available, the risk mitigation plan may compensate by enhancing other aspects of hazard controls such as training, PPE, and workplace practices.

Once dispensed, the IP must be prepared for transport to the clinic, which may include transport offsite. Transfer of the IP from the pharmacy to the clinic should be performed utilizing sealed, leakproof, and labeled primary and secondary containment. Oftentimes, secondary containment is in the form of sealed specimen bags containing the biohazard symbol. The route taken to the clinic should minimize potential exposure to patients in the event a spill takes place. If the IP is transported or shipped to a clinic offsite, it should be packaged and transported/shipped in a manner compliant with DOT/IATA requirements.

The IP is administered in facilities that may vary based on the type of clinical trial but may include examination rooms, operating rooms, and infusion suites. Biosafety practices for clinical facilities are reviewed elsewhere.^81,82^

### Collaboration Between the IBC and the Institutional Review Board to Protect Subjects, Community, and Environment

Part of the review by an Institutional Review Board (IRB) as outlined by The Common Rule (45 CFR 46)^83^ entails weighing whether the benefits to the research subject and society are worth the risks borne by the research subject and if the risks are adequately minimized. The study's inclusion and exclusion criteria are designed to maximize benefits to subjects and the research whereas minimizing risks to the research subjects.

Research subjects must be provided with informed consent that adequately describes the experimental procedures and the risks associated with participating in the research. Waiver can be sought for minimal risk research deemed to be no greater than ordinarily encountered in the daily life of the general population or during the performance of routine physical or psychological examinations or tests.^84^ It is extremely unlikely that a clinical trial involving genetically modified organisms or recombinant/synthetic nucleic acid molecules would be exempted from informed consent.

The IBC can provide guidance to the IRB in review of the informed consent to ensure the risks associated with the genetically modified microorganisms are adequately described. If concerns exist about at home care, shedding, exposure to others and the environment, or waste treatment and disposal, such information can be provided through additional subject training materials. Subject facing documents should be reviewed by the IRB.^84^

### Regulatory Considerations

We previously reviewed the regulatory review process for clinical trials. With respect to NIH Guidelines, clinical trials fall under Section III-C-1, Experiments Involving the Deliberate Transfer of Recombinant or Synthetic Nucleic Acid Molecules, or DNA or RNA Derived from Recombinant or Synthetic Nucleic Acid Molecules, into One or More Human Research Participants, requiring IBC approval before initiation.^85^ Whereas Section III-F of NIH Guidelines grants exemptions for some research involving recombinant bacteria or bacteriophages, Section III-F1 states, “If a synthetic nucleic acid is deliberately transferred into one or more human research participants and meets the criteria of Section III-C, it is not exempt under this Section.”

Concerns over Dual Use Research of Concern (DURC) most often do not apply to clinical trials as the Federal policy is currently limited to 15 highly virulent select agents or toxins.^86^ The attenuated strains likely used in clinical trials will most likely be excluded from the DURC policy. However, the IBC may consider whether the genetic manipulations could constitute a risk as outlined by the seven experiments of concern (listed hereunder).

Seven experiments of concern as outlined in the Federal DURC policy:

(a) Enhances the harmful consequences of the agent or toxin
(b) Disrupts immunity or the effectiveness of an immunization against the agent or toxin without clinical or agricultural justification
(c) Confers to the agent or toxin resistance to clinically or agriculturally useful prophylactic or therapeutic interventions against that agent or toxin or facilitates their ability to evade detection methodologies
(d) Increases the stability, transmissibility, or the ability to disseminate the agent or toxin
(e) Alters the host range or tropism of the agent or toxin
(f) Enhances the susceptibility of a host population to the agent or toxin
(g) Generates or reconstitutes an eradicated or extinct agent or toxin listed in Section (III.1) above.

Environmental release or interpersonal spread of recombinant bacteria can arise from shedding in feces for orally administered products or from contact with skin for those topically administered. Environmental release or transmission to others should also be considered for studies designed to involve self-administration of recombinant bacteria by untrained research subjects.

Although the FDA considers the need for an environmental impact assessment with each IND application, most IPs are granted a categorical exclusion at that stage, and environmental impacts are fully considered only when the FDA receives a biologics license application for FDA approval. This typically occurs when strong safety and efficacy data are submitted at the end of phase III clinical trials.^87^

“In the event FDA action on an IND would increase the use of a drug, the agency's experience has demonstrated that significant environmental effects would not occur because the investigational use is limited and controlled” 8 (62 FR 40570 at 40578). In addition, the preamble to the Final Rule went on to state, “In the event FDA has reason to believe its action on an IND may significantly affect the environment, FDA will invoke the provision relating to ‘extraordinary circumstances' and require an EA.” (Id. at 40579) The threshold for “significantly” affecting the quality of the environment is defined under 40 CFR 1508.27 as follows:

- The degree to which the effects of the IP on the quality of the environment are likely to be highly controversial [40 CFR 1508.27(b)(4)].
- The degree to which the possible effects of the IP on the human environment are highly uncertain or involve unique or unknown risks [40 CFR 1508.27(b)(5)].
- The degree to which the IP may adversely affect an endangered or threatened species or its habitat that has been determined to be critical under the Endangered Species Act of 1973 [40 CFR 1508.27(b)(9)].
- Whether the effects of the IP on the environment threaten a violation of Federal, State, or local law or requirements imposed for the protection.

## Overview of Select Clinical Trials and a Marketed LBP

**Figure 4.**
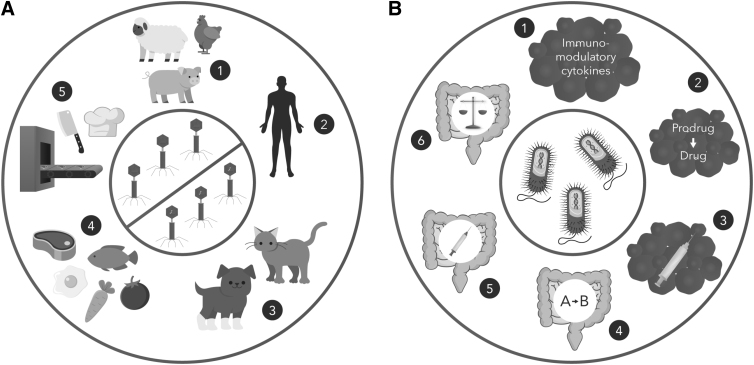
Uses of bacteriophages and recombinant bacteria. Some of these applications are currently approved in the United States, whereas others are under investigation in clinical trials. **(A)** Use of wild-type or recombinant bacteriophages. (1) Treatment of bacterial infections in farm animals, (2) treatment of bacterial infections in humans, (3) treatment of bacterial infection in pets, (4) reducing bacterial contamination of food, and (5) prevention of bacterial contamination in food preparation. **(B)** Use of recombinant bacteria in medicine. (1) Directing the production of immunomodulatory cytokines in tumors to stimulate an immune response, for example, IL-12, IFN-α, and IFN-β, (2) converting prodrugs to active drugs in tumors, (3) production of tumor antigens to elicit an anticancer immune response, (4) expressing enzymes that breakdown pathological compounds in the intestines, (5) expressing antigens in the intestines to induce immune responses to infectious agents, and (6) correcting imbalance of the microbiota in dysbiosis. IL, interleukin.

Relative to virus-based IPs, there has been a limited number of clinical trials involving recombinant bacteria for a variety of indications, which we summarize here (Figure 4 and Tables 2 and 3) and are reviewed further elsewhere.^88^ These bacteria have distinguishing characteristics that require consideration by IBCs and biosafety professionals. Most benignly are GRAS lactic acid bacilli (LAB), which present minimal biosafety issues, as long as the transgenes are benign.

In the middle are bacteria with moderate biosafety concerns, such as *Escherichia coli* Nissle 1917 and *Bifidobacterium longum*. Finally, there are pathogens, either opportunistic or overt, which have been engineered for therapeutic purposes. This latter group merits the closest scrutiny since a failure of the safety systems or release of the bacteria into the environment would be detrimental.

The first recombinant bacterium to be examined in clinical trials was *Lactobacillus lactis* encoding the gene for the immunosuppressive cytokine interleukin-10 (IL-10) in 2006 (LL-Thy12).^89^ The study's aim was determining whether the bacteria could reduce inflammation from Crohn's disease. *L. lactis* is a member of the LAB group of bacteria that are normal constituents of the human microbiota and are considered GRAS and safe for administration to people. The *L. lactis* strain had the *thyA* gene replaced with that of IL-10, so these bacteria were replication-defective.

Other representative LAB used in clinical trials included *L. lactis* strain sAGX0085 encoding human trefoil factor 1, otherwise known as AG013,^90^ which possesses wound healing properties. The live recombinant bacteria were administered as a mouthwash to patients with oral mucositis from chemoradiation in 2013, but the study was terminated because of lack of efficacy. A 2018 study involved *L. lactis* secreting human proinsulin and IL-10, also known as AG019, administered orally through a capsule to patients with type 1 diabetes to suppress the autoimmune response that damages insulin-producing cells of the pancreas causing the disease.^91^ The recombinant proteins were designed to act after colonization of the intestinal tract by the bacteria.

Although not a LAB, *B. longum* is an obligate anaerobic gram-negative rod that is a normal member of the human intestinal microbiota. It has been engineered for a couple of therapeutic uses that have made their way to clinical trials. APS001F is *B. longum* genetically engineered to express cytosine deaminase.^92^ Cytosine deaminase activates the prodrug flucytosine into the chemotherapeutic 5-fluorouracil to render it active against cancer cells by inhibiting synthesis of DNA and RNA.

Because APS001F is an obligate anaerobe, upon injection into the patient, it will home to and proliferate in the hypoxic tumor environment. *B. longum* has also been developed into a true gene therapy vector for the treatment of cancer. bacTRL-IL-12 is endowed with a plasmid encoding IL-12, an immunostimulatory cytokine with beneficial effects in fighting cancer. The bacteria are engineered to deliver the plasmid to host cells in the tumor microenvironment to enable them to locally produce IL-12.

A workhorse probiotic strain for recombinant use is *E. coli* strain Nissle 1917 isolated in 1917 by Alfred Nissle.^93^ It is marketed in Europe and Canada with the trade name Mutaflor as a probiotic with numerous putative health benefits, including antibacterial and anti-inflammatory activities for treating inflammatory bowel diseases. It is not approved for marketing in the United States. It is noteworthy that, even though it was isolated from a human, it does not colonize the intestinal tract very well, necessitating frequent dosing.

The strain's poor ability to colonize serves as a risk mitigation feature, as any unpredicted adverse effects should be short lived. As an *E. coli* strain, genetic manipulations are easy to perform. The strain's major drawback is that it encodes a toxin, colibactin, which causes DNA damage and has been linked to intestinal cancer in some animal models.^93^ One could argue that deleting the colibactin genes would render Nissle 1917 safer; however, there is evidence that colibactin contributes to the beneficial probiotic activity of the strain.^94^ There have been several clinical trials using engineered Nissle 1917 and several more that are ongoing.

The Nissle 1917-based strain SYNB1934 has been engineered to express phenylalanine-degrading enzymes for the treatment of phenylketonuria.^95^ Although resident in the intestines, the bacteria will consume phenylalanine from the diet and prevent the toxic accumulation of this amino acid in these patients lacking phenylalanine-degrading pathways. SYNB1934 has been rendered replication defective by deletion of the *dapA* gene. As noted earlier, in the absence of DAP, the bacteria will lyse, thereby releasing the enzymes into the intestinal milieu.

Another Nissle 1917 derivative, SYNB1020, was engineered to convert ammonia to arginine.^96^ It is aimed at decreasing intestinal uptake of ammonia in patients with hyperammonemia, a disease in which ammonia is not correctly metabolized by the liver resulting in liver damage and neurocognitive dysfunction. The recent clinical trial was halted due to lack of efficacy.

SYNB8802 is Nissle 1917 engineered to metabolize enteric oxalate to treat hyperoxaluria.^97^ Hyperoxaluria is caused by the body producing or absorbing too much oxalate, which is an end product of metabolism and present in many plant foods. Excess levels of oxalate in urine can cause kidney stones. SYNB8802 contains genes to uptake and degrade oxalate, and its replication is inhibited by deletion of the *thyA* gene.

SYNB1891 represents a very different use of Nissle 1917. It is designed to stimulate an immune response against solid tumors by activation of the STING pathway in immune cells to turn on innate immune pathways.^98^ Cyclic di-nucleotides bind to and activate STING, causing the production of type I interferons (IFN-α and IFN-β). SYNB1891 encodes diadenylate cyclase (*dacA*) from *L. monocytogenes* to produce cyclic di-nucleotides, and the expression of *dacA* was engineered to be induced under the hypoxic conditions of a solid tumor. Therefore, unlike the preceding Nissle 1917 strains, SYNB1891 is injected intratumorally, rather than ingested.

At the higher end of the risk spectrum for recombinant bacterial therapeutics is *L. monocytogenes*, a RG2 pathogen notorious for contaminating meat and cheese, causing abortion, neonatal meningitis, and infections of the immunocompromised. However, this pathogen possesses some characteristics that make it a useful candidate for therapy of certain diseases. It invades host cells and escapes the endosome/phagosome into the cytoplasm before killing host cells. When infecting antigen presenting cells, it can stimulate CD8 cytotoxic T lymphocyte response through presentation of antigens through major histocompatibility (MHC) class I and CD4 Th1 cell-mediated immune response through MHC II.^99^

ADXS-503 is *L. monocytogenes* that has been engineered to express 22 different tumor antigens commonly found in patients with metastatic squamous and non-squamous cell lung cancer (NSCLC).^64^ ADXS11–011 is *L. monocytogenes* engineered as a vaccine against human papilloma virus (HPV) cancers by encoding the E7 oncoprotein.^100^ ADXS11-011 was attenuated by mutation of *prfA*, which encodes a central essential virulence regulatory protein. It was evaluated in at least five clinical trials for HPV-induced cervical, oropharyngeal, and anal cancer.

ADXS31–164 is *L. monocytogenes* expressing the chimeric HER2/neu tumor-associate antigen for induction of antitumor immunity in solid tumors expressing HER2.^101^ This strain is interesting for two reasons. First, it is attenuated by deletion of the *actA* virulence gene. Second, it uses a balanced lethal system to select for the recombinant plasmid encoding the transgene. The *dal* and *dat* genes, necessary for synthesis of the essential peptidoglycan component d-alanine, were deleted. Providing the *dal* gene on the plasmid restores d-alanine synthesis and viability. ADXS31–164 is currently in clinical trials.

ADXS-NEO is an interesting derivate of the *L. monocytogenes*-based cancer vaccines in that it is engineered to express patient-specific tumor antigens.^102^ CRS-207 is *L. monocytogenes* that expresses mesothelin, a tumor-associated antigen associated with malignant pleural mesothelioma and pancreatic cancer. It has been examined in several clinical trials in combination with chemotherapy with mixed results.^103^

JNJ-64041757 is *L. monocytogenes* that is attenuated by deletion of the actin assembling protein *actA* and internalin B (*inlB*) virulence genes.^104^ The ActA protein facilitates trafficking of *L. monocytogenes* through the cytosol for transmission to neighboring cells, and internal B is necessary for *L. monocytogenes* entry into non-phagocytic cells. Thus, deleting both *actA* and *inlB* restricts *L. monocytogenes* to initially infected antigen presenting cells. JNJ-64041757 was engineered to express mesothelin, a tumor-associated antigen of NSCLC. In phase 1 and 2 clinical trials, patients were injected intravenously along with a checkpoint inhibitor to increase the immune response. Although the treatment was safe, it was not effective.

Another overt RG2 bacterial pathogen that has been adapted into cancer treatment through genetic engineering is *Salmonella* Typhi. Typhoid fever is a systemic disease mostly in the developing world caused by this pathogen that is related to the ubiquitous *Salmonella* Typhimurium that causes diarrhea. Efforts at creating a live attenuated typhoid fever vaccine led to the creation of Ty21a. This strain was generated in 1975, not by recombinant methods that would be the standard today, but by chemical mutagenesis.^105^ However, it has proven safe and effective, and is licensed and used worldwide and was recently FDA-approved as Vivotif. Of note, the vaccine is capable of stimulating all branches of the immune system (mucosal, humoral, and cell-mediated), and so it represents an ideal platform for stimulating immune responses for a variety of purposes.

Strain VXM01 is Ty21a that has been engineered to express human vascular endothelial growth factor receptor 2 (VEGFR-2). Solid tumors require increased vasculature to provide nourishment, and so they stimulate neovascularization by inducing vascular endothelial growth factor (VEGF) production. VEGFs bind to VEGFRs, and some tumors stimulate VEGFRs on the local vascular endothelium. By stimulating an immune response to the VEGFR-expressing tumor-associated cells, the blood supply to the tumor can be cut off. In initial clinical trials for stage IV pancreatic cancer, orally administered VXM01 was safe and demonstrated some efficacy in stimulating cell-mediated immunity against VEGFR.^106^

*Salmonella* Typhimurium is also receiving attention as an oncolytic and cancer vaccine effector.^107^ It thrives in the hypoxic tumor environment and thus preferentially infects these sites. Similar to *Listeria*, it is an intracellular bacterium. It can induce apoptosis in infected tumor cells, compete for nutrients, and limit angiogenesis, which is critical for tumor growth. An interesting application of *Salmonella* Typhimurium was engineering it to express *E. coli* cytosine deaminase (strain TAPET-CD) so that it would activate the chemotherapy pro drug 5-fluorocytosine in situ.^108^ The patients in the initial clinical trial experienced the intended drug activation, but the severity of their disease precluded efficacy.

Two clinical trials have been done/are ongoing using *Salmonella* Typhimurium expressing IL-2 to stimulate the immune response in the tumor environment. Results of the first trial with SalpIL2 have not been published. However, for Saltikva, preliminary results presented at the annual meeting of the American Association for Cancer Research earlier in 2023 demonstrated some efficacy and promise.^109^

As mentioned hereunder, a very unique use of recombinant bacteria in cancer treatment is *B. longum* product, bacTRL-IL-12, which actually delivers a recombinant plasmid encoding IL-12 to host cells in the tumor environment to stimulate the immune system. It was investigated in clinical trials.

This discussion of the use of recombinant bacteria as probiotics and therapeutics would not be complete without mentioning a product that seemingly has escaped government oversight and is marketed online in the U.S. Zbiotics is based on *Bacillus subtilis*, a common nonpathogenic gram-positive rod that is the *E. coli* K12 equivalent in the gram-positive world (zbiotics.com).^24^ The bacteria have been genetically engineered to express aldehyde dehydrogenase, an enzyme that degrades acetaldehyde, the toxic end product of ethanol metabolism and a major source of the symptoms of hangover after overconsumption of alcohol.

Zbiotics is taken orally prophylactically in anticipation of overdrinking, and somehow the presence of the acetaldehyde-degrading bacteria in the intestines prevents the well-known side effects of overdrinking. The web page of the parent company states “ZBiotics is fully FDA-compliant for safety and adheres to all regulatory requirements for sale in the U.S. Note that FDA compliance is not FDA approval.” (zbiotics.com). The outcome of this commercial foray into recombinant probiotics will be interesting.

### Recombinant Bacteriophage as Antibacterial Therapy

Because bacteriophages are viruses that kill bacteria, they are being investigated as antibiotic alternatives in the age of rampant antibiotic resistance.^18^ There are three uses of phages under development for human health. The oldest is the use of WT phages to treat bacterial infections.^18^ More recently, WT phages have been approved for use on food products to reduce pathogenic bacterial contamination. Finally, phages are being genetically engineered to improve their antibacterial properties.

In the realm of food safety, in 2006 the FDA approved the phage product ListShield^110^ for use on meat and poultry to reduce *L. monocytogenes* contamination. There are now several FDA-approved phage products for treatment of food.^111^

Phage therapy, as it is called, was actually initiated about 100 years ago in Europe and was practiced in the United States for some time until it fell out of favor from failure and the advent of antibiotic therapy. However, phage therapy has been continually used in Eastern Europe to this day and is experiencing a renaissance in the United States as research has resumed. This therapeutic use of WT phages would fall outside of the purview of the IBC, since there is no recombinant DNA involved. However, a Danish company, SNIPR BIOME, has developed recombinant bacteriophages aimed at attacking and eliminating specific bacteria from the microbiota.^112^ Since phages are very specific in their host ranges, they can be used to target specific species.

To improve on the natural killing activity of phages, SNIPR BIOME, engineered into some *E. coli* phages CRISPR-Cas machinery that is aimed at cleaving the host *E. coli* genome and killing the cell. The interesting twist in this story is that CRISPR-Cas is a bacterial system that evolved to inactivate incoming phage DNA. A pool of recombinant bacteriophage was engineered to recognize most *E. coli* strains and encode an *E. coli*-specific CRISPR system. The intended use of this product, SNIPR001, is to clear the intestinal tract of patients who are about to undergo chemotherapy for cancer. *E. coli* is a leading cause of sepsis in these patients, as the integrity of their intestinal mucosal and their immune system are compromised.

Although phages are benign, since they cannot infect animal cells, the IBC consideration here is biocontainment. Since the goal and major advantage of such phage therapy is for the phages to reproduce immensely as they infect and kill their bacterial hosts, the phages cannot be engineered to be conditionally or non-replicative. If phages are going to be administered orally to treat the intestinal tract, they will be excreted into the environment and sewage system, where they will, no doubt, encounter *E. coli* hosts. This represents a deliberate release of recombinant material that, although not technically viable, is capable of replication.

Other considerations in devising phages for clinical, agricultural, veterinary, or industrial use are screening them for detrimental properties that are inherent to some phages. For example, several bacterial toxins, for example, cholera toxin, diphtheria toxin, and botulinum toxin, are encoded by phages.^18^ Some phages, temperate phages, are capable of stably coexisting with their bacterial hosts, rather than always lysing them. Not only would temperate phages not be effective at killing the targeted bacteria, but they could also participate in genetic exchange through a process called specialized transduction^18^ Fortunately, these characteristics can be identified through sequencing the phage genome.

## Future Prospects

Advancement in the use of genetically modified bacteria is showing promise in preclinical oncology research,^113^ which can result in innovative applications for clinical research. Since obligate and facultative anaerobes traffic to the hypoxic tumor microenvironment, they exhibit potential for targeted therapeutic delivery that is not seen in systemic chemotherapy.^39^ Bacteria can be further targeted by expressing antibodies or ligands for tumor-associated antigens.^114^ Potential applications for bacterial homing and colonization of the tumor microenvironment include oncolytics, immune modulation, and delivery of therapeutic substances.

Bacteria-derived molecules such as peptidoglycan, lipopolysaccharide, and lipoteichoic acid inherently possess immune stimulatory properties.^115^ We previously mentioned *B. longum* that has been engineered for different uses such as expression of cytosine deaminase to convert the chemotherapeutic prodrug flucytosine into the chemotherapeutic 5-fluorouracil to render it active against cancer cells by inhibiting synthesis of DNA and RNA. *B. longum* has also been developed into a true gene therapy vector (bacTRL-IL-12), which delivers a plasmid encoding the immunostimulatory cytokine IL-12 to the tumor microenvironment.

Bacteria have been engineered to secrete a number of immune modulating cytokines or chemokines to recruit white blood cells,^102,116–121^ or checkpoint inhibitor antibodies to interfere with immune suppression. A recently published study showed modified *E. coli* trafficked to solid tumors and secreted synthetic antigens, which could be used to home probiotic guided chimeric antigen receptor T cells (Pro-CAR) to the tumor microenvironment and causes tumor cell lysis.^122^

If Pro-CAR successfully translate to clinical trials, they may aid CAR T cells in demonstrating efficacy against solid tumors. Although CAR T cells have been successful against leukemias and lymphomas, they have struggled to demonstrate efficacy in the immunosuppressive microenvironment of solid tumors.

Assuming that recombinant bacterial LBPs are approved for human use, the question arises if the intended patients will accept the therapy. Genetically modified organisms in foods have met with extreme resistance, as there is widespread fear and lack of trust in both the biotechnology industry and government regulators. It is possible that treating human disease will be viewed as more acceptable, since the benefits of the treatments would engender more compassion. The patients, themselves, who likely would have suffered failure of standard treatments, if available, are more likely to consider new forms of therapy.

As noted earlier, there are already two genetically engineered live attenuated bacterial vaccines that are FDA-approved for cholera and typhoid fever. So, in one sense, the bridge of administering recombinant bacteria for medical purposes has already been crossed. With the number and variety of recombinant bacteria in clinical trials, as well as preclinical development, it is clear that this realm of medicine is on the precipice of rapid and massive expansion, much as is the case for viral vectored gene therapy. It will, therefore, be critical for IBCs to prepare accordingly.

## Summary

Use of bacteria and bacteriophages as IPs is resurgent, and discussion of the risks associated with genetic modification of these organisms has become pertinent to the biosafety community. Bacteria are being developed as recombinant LBPs, therapeutics, gene therapy agents, and live vaccines. They have been used in clinical trials for a wide variety of diseases, but the only strain to receive FDA approval to date is the Vaxchora cholera vaccine. There are numerous preclinical studies generating a pipeline of new agents destined for clinical trials.

In this review, we summarized clinical trials conducted to date involving recombinant bacteria and outlined the process for conducting risk assessments for these agents. Because bacteria differ from the viral gene therapy vectors that are most commonly utilized for gene therapy, they present special challenges to IBCs that will be charged with the oversight of their preclinical and clinical development and study. Bacteria are more complex than viruses, particularly with regard to control of replication.

Orally administered live therapeutics represent a special challenge for environmental release. Recombinant live attenuated bacterial vaccines are already in use, and a recombinant LBP is already being marketed in the United States outside of FDA oversight. Therefore, it is critical that IBCs acquire the necessary expertise in bacteriology, especially genetics and physiology, as well as immunology and environmental microbiology.
